# *Gardnerella vaginalis* and *Neisseria gonorrhoeae* Are Effectively Inhibited by Lactobacilli with Probiotic Properties Isolated from Brazilian Cupuaçu (*Theobroma grandiflorum*) Fruit

**DOI:** 10.1155/2021/6626249

**Published:** 2021-04-29

**Authors:** Nathan das Neves Selis, Hellen Braga Martins de Oliveira, Yan Bento dos Anjos, Hiago Ferreira Leão, Beatriz Almeida Sampaio, Thiago Macêdo Lopes Correia, Mariane Mares Reis, Thamara Louisy Santos Brito, Carolline Florentino Almeida, Larissa Silva Carvalho Pena, Laís Ferraz Brito, Roberta Maria Ornelas, Tizá Teles Santos, Guilherme Barreto Campos, Jorge Timenetsky, Mariluze Peixoto Cruz, Andréa Miura da Costa, Regiane Yatsuda, Ana Paula Trovatti Uetanabaro, Lucas Miranda Marques

**Affiliations:** ^1^Programa de Pós-Graduação em Biologia e Biotecnologia de Microrganismos, Pavilhão Max de Menezes, Universidade Estadual de Santa Cruz, Campus Soane Nazaré de Andrade, Salobrinho, Rodovia Jorge Amado, Km 16, CEP 45662-900 Ilhéus, BA, Brazil; ^2^Instituto Multidisciplinar em Saúde, Universidade Federal da Bahia, Campus Anísio Teixeira, Rua Hormindo Barros, 58, CEP 45029-094 Vitória da Conquista, BA, Brazil; ^3^Departamento de Ciências Biológicas, Laboratório de Microbiologia da Agroindústria, Universidade Estadual de Santa Cruz, Campus Soane Nazaré de Andrade, Salobrinho, Rodovia Jorge Amado, Km 16, CEP 45662-900 Ilhéus, BA, Brazil; ^4^Instituto de Ciências Biomédicas, Universidade de São Paulo, Avenida Professor Lineu Prestes, 2415, CEP 05508-900 São Paulo, SP, Brazil

## Abstract

In recent years, certain *Lactobacillus* sp. have emerged in health care as an alternative therapy for various diseases. Based on this, this study is aimed at evaluating in vitro the potential probiotics of five lactobacilli strains isolated from pulp of cupuaçu fruit fermentation against *Gardnerella vaginalis* and *Neisseria gonorrhoeae*. Our lactobacilli strains were classified as safe for use in humans, and they were tolerant to heat and pH. Our strains were biofilm producers, while hydrophobicity and autoaggregation varied from 13% to 86% and 13% to 25%, respectively. The coaggregation of lactobacilli used in this study with *G. vaginalis* and *N. gonorrhoeae* ranged from 15% to 36% and 32% to 52%, respectively. Antimicrobial activity was present in all tested *Lactobacillus* strains against both pathogens, and the growth of pathogens in coculture was reduced by the presence of our lactobacilli. Also, all tested lactobacilli reduced the pH of the culture, even in incubation with pathogens after 24 hours. The cell-free culture supernatants (CFCS) of all five lactobacilli demonstrated activity against the two pathogens with a halo presence and CFCS characterization assay together with gas chromatography revealed that lactic acid was the most abundant organic acid in the samples (50% to 62%). Our results demonstrated that the organic acid production profile is strain-specific. This study revealed that cupuaçu is a promising source of microorganisms with probiotic properties against genital pathogens. We demonstrated by in vitro tests that our *Lactobacillus* strains have probiotic properties. However, the absence of in vivo tests is a limitation of our work due to the need to evaluate the interaction of our lactobacilli with pathogens in the vaginal mucosa. We believe that these findings may be useful in developing a product containing our lactobacilli and their supernatants in order to support with vaginal health.

## 1. Introduction

The World Health Organization (WHO) describes probiotics as live microorganisms that, when administered in adequate amounts, confer health benefits to the host [1]. Regarding women's health, probiotics have already shown that they can assist in recovering vaginal homeostasis. Generally, results from traditional therapies are optimized when associated with these microorganisms [2]. The benefits attributed to probiotics are related to (a) production of organic acids, (b) modulating the composition of the intravaginal microbiota [3], (c) antioxidant activity and reducing damages caused by oxidation [4], (d) effects on the functionality of the mucosal and systemic immune systems, (e) reducing anti-inflammatory reactions, (f) competition with pathogens [5], and (g) production of hydrogen peroxide and bacteriocins [6].

In the last decade, health professionals have become increasingly interested in new treatment alternatives as a result of consumer demands for better therapies, due to problems resulting from antibiotic resistance and undesirable side effects [7]. Reports from the literature show that lactobacilli isolated from functional foods demonstrate promising probiotic effects beneficial to human health. A number of studies focused on new strains that have probiotic characteristics illustrate that the beneficial properties are strain-specific and can act on different pathogens [8–11]. The administration of probiotics that colonize the vaginal tract can be important in maintaining normal urogenital health and also preventing and treating infections [12], such as bacterial vaginosis (BV) and gonorrhea.

BV is the most commonly reported microbiological syndrome among women of childbearing age, where the genital microbiota changes from the dominant *Lactobacillus* sp. to a polymicrobial anaerobic population [13]. Currently, with uncertain pathogenesis, BV is no longer considered an infection but rather as a dysbiosis, a microbial imbalance in the vaginal microbiota. Many microbial pathogens have been associated with BV; however, *Gardnerella vaginalis* is the bacteria most related to this clinical condition [14]. BV recurrence rates are high in symptomatic women who present vaginal malodor, discharge, itching, and increased vaginal pH [15]. BV is related to obstetric and gynecological complications, including pelvic inflammatory disease, premature labor, and increased incidences of sexually transmitted infections, such as cervicitis by *Neisseria gonorrhoeae*, bacteria that causes gonorrhea [16].

Gonorrhea is a sexually transmitted infection of global proportions associated with pelvic inflammatory disease, infertility, urethritis in men, and mucopurulent cervicitis in women [17]. The gonococci colonize the genital, anal, ocular, and nasopharyngeal mucosa. With a wide variety of niches, *N. gonorrhoeae* expresses a repertoire of factors that allow its replication, survival and modulation, and evasion of the host immune system [18]. In recent years, even with effective antibiotics, gonorrhea remains a serious and common public health problem, and the emergence of resistant strains has hampered and limited treatment options [19]. Thus, considering that researchers around the world have sought alternative solutions against genital pathogens of medical importance, our study is aimed at evaluating in vitro the potential probiotic effects of five *Lactobacillus* strains isolated from cupuaçu (*Theobroma grandiflorum*) fruit fermentation against *G. vaginalis* and *N. gonorrhoeae*.

## 2. Material and Methods

### 2.1. Microorganisms and Growth Conditions

*Lactobacillus* sp. were isolated from originate spontaneous pulp of cupuaçu (*Theobroma grandiflorum*) fruit fermentation from Ilhéus and Itabuna region, Bahia, Brazil, and donated by the Laboratory of Applied Microbiology from the State University of Santa Cruz, Brazil [20]. The five lactobacilli strains used in this study were *Lactobacillus casei* (Lc24), *Lactobacillus fermentum* (Lf38 and Lf47), and *Lactobacillus plantarum* (Lp81 and Lp90). The lactobacilli strains were grown in de Man, Rogosa, and Sharpe (MRS) agar or broth (Acumedia, Lansing, USA) for 18 to 24 hours, at 37°C, under microaerophilic conditions (5% CO_2_ atmosphere)—overnight cultures.

*Gardnerella vaginalis* ATCC 49154 was grown on 5% blood agar (HiMedia Laboratories, Mumbai, India) or Brain and Heart Infusion (BHI) broth (HiMedia) for 18 to 24 hours, at 37°C, under microaerophilic conditions (5% CO_2_ atmosphere)—overnight culture.

*Neisseria gonorrhoeae* (clinical isolate) was grown on chocolate agar (HiMedia) or BHI broth for 18 to 24 hours, at 37°C, under microaerophilic conditions (5% CO_2_ atmosphere)—overnight culture.

### 2.2. Preparation of Cell-Free Culture Supernatant (CFCS)

The assay for obtaining the CFCS was adapted from Pessoa et al. [21]. After overnight cultures of *Lactobacillus* strains were centrifuged (3,000 × *g*, 15 min), the supernatants were discarded; cell pellets (lactobacilli) were washed twice with sterile saline (0.9% NaCl) and resuspended to a final concentration (10^8^ CFU.mL^−1^). After this, suspensions (1.5 mL) of each *Lactobacillus* sp. were inoculated (10%, *v*/*v*) in sterile MRS broth (15 mL), and after incubation (24 h, 37°C, 5% CO_2_ atmosphere), the cultures were centrifuged (3000 × *g*, 15 min); supernatants were aspirated using sterile syringes and sterilized by filtration (0.22 *μ*m nitrocellulose filter; Merck, Darmstadt, Germany) for obtaining CFCS.

### 2.3. Technological Evaluation of Lactobacilli

#### 2.3.1. Heat Tolerance Assay

Heat resistance of our lactobacilli strains was evaluated according to Paéz et al. [22] with modifications. Initially, suspensions (10^8^ CFU.mL^−1^) of lactobacilli strains were obtained as previously described. An aliquot (100 *μ*L) was resuspended in volume (500 *μ*L) of 10% skim milk (Nestlé, Araçatuba, Brazil). Then, each cell suspension was incubated in a water bath (60°C, 5 min), followed by cooling in an ice bath. Aliquots (10 *μ*L) of each strain were plated on MRS agar, and after incubation (48 h, 37°C, 5% CO_2_ atmosphere), the colonies were counted and enumerated considering CFU.mL^−1^. As a control, aliquots (10 *μ*L) of the same samples were plated under the same conditions before exposure to heat.

#### 2.3.2. pH Tolerance Assay

The analysis of bacterial growth under varied pH was adapted from Melo et al. [10]. MRS broth solutions of pH 3, pH 4, pH 5, pH 6, pH 7, and pH 8 were prepared by addition of 1 mol.L^−1^ of hydrochloric acid or 1 mol.L^−1^ of sodium hydroxide. The trials were performed in 96-well microplates, where in 180 *μ*L of MRS broth at each pH was inoculated with 20 *μ*L of active culture (10^8^ CFU.mL^−1^) or saline as a control. The microplate was incubated overnight, and the optical density (600 nm) was determined every 8 hours using a spectrophotometer (Tp-reader, Thermoplate, USA).

### 2.4. Safety Evaluation of Lactobacilli

#### 2.4.1. Antibiotic Susceptibility Assay

Susceptibility of *Lactobacillus* strains to antimicrobials was determined by the modified agar diffusion method of Clinical and Laboratory Standards Institute (CLSI). Overnight cultures of *Lactobacillus* strains were adjusted to 0.5 McFarland standards. Then, an aliquot of this suspension was swabbed onto MRS agar plates, followed by the arrangement of antibiotic disks. Plates were incubated overnight, and the diameters of the halos were measured and classified as sensitive (S), moderately sensitive (MS), and resistant (R), according to Charteris et al. [23]. The antimicrobials (Laborclin, Pinhais, Brazil) tested were ampicillin (10 *μ*g), ceftriaxone (30 *μ*g), ciprofloxacin (5 *μ*g), clindamycin (2 *μ*g), chloramphenicol (30 *μ*g), erythromycin (15 *μ*g), nitrofurantoin (300 *μ*g), penicillin (10 *μ*g), and vancomycin (30 *μ*g).

#### 2.4.2. Hemolytic Activity Assay

The hemolytic activity assay was adapted from Abouloifa et al. [9]. Initially, suspensions (10^8^ CFU.mL^−1^) were spot-inoculated (10 *μ*L) on 5% blood agar. After incubation (48 h, 37°C, 5% CO_2_ atmosphere), the hemolytic activity was detected by observing a clear zone of hydrolysis around the colonies (*β*-hemolysis), partial hydrolysis with green-hued zones around colonies (*α*-hemolysis), or no zone around colonies (*γ*-hemolysis). The *γ*-hemolysis was considered negative hemolysis.

### 2.5. Evaluation of the Cell Surface Properties of Lactobacilli

#### 2.5.1. Biofilm Formation Assay

Biofilm formation assay was adapted from Ouarabi et al. [24]. Initially, an aliquot (10 *μ*L) of each *Lactobacillus* (10^8^ CFU.mL^−1^) was inoculated in MRS broth (200 *μ*L) in a 96-well polystyrene plate and then incubated overnight. After incubation, the plate was washed twice with sterile saline to remove nonadherent cells. The cells were fixed with 96% ethanol (200 *μ*L) and incubated (15 min, room temperature). After this, the plates were emptied and then filled with violet crystal (200 *μ*L, 0.1%) and incubated (15 min, room temperature). Then, the plate was washed twice with sterile saline, and the wells were resuspended with 96% ethanol (200 *μ*L). The absorbance (650 nm) of the samples was immediately measured and taken as an indication of biofilm formation. Sterile medium was included as a negative control to ensure that the influence on biofilm formation was not attributed to a nonspecific binding effect to the violet crystal. Based on the optical densities of the isolates (DO_*I*_) and the negative control (DO_*C*_), the formation of biofilm by lactobacilli was classified according to their adherence: nonadherent, DO_*I*_ ≤ DO_*C*_; weakly adherent, DO_*C*_ < DO_*I*_ ≤ (2 × DO_*C*_); moderately adherent, (2 × DO_*C*_) < DO_*I*_ ≤ (4 × DO_*C*_); and strongly adherent, (4 × DO_*C*_) < DO_*I*_.

#### 2.5.2. Hydrophobicity Assay

The hydrophobicity of the *Lactobacillus* strains was verified by testing microbial adherence to hydrocarbons (MATH), using a method adapted from Rodríguez et al. [25], and using xylene as solvent. Initially, suspensions (10^8^ CFU.mL^−1^) of lactobacilli strains were measured (OD 660 nm). The solvent (xylene, 0.4 mL) was then added to each bacterial suspension (1 mL), and the mixtures were vortexed vigorously and incubated (2 h, 37°C). Then, the lower aqueous phase was carefully removed and read in a spectrophotometer. The percentage of hydrophobicity was calculated using Equation (1), where *A*_0_ indicates the absorbance at time 0 hour and *A*_2_ indicates the absorbance after 2 hours. MATH can be classified as low (MATH < 33%), medium (33% < MATH < 66%), or high (MATH > 66%). The hydrophobicity can also be presented as microbial adhesion to solvents (MATS), classifying the bacterial surface as hydrophobic (MATS ≥ 55%), amphiphilic (45% ≤ MATS ≤ 55%), or hydrophilic (MATS ≤ 45%).

(1) $$\begin{array}{c} H\%=\frac{A0-A1}{A0}\times 100. \end{array}$$

#### 2.5.3. Autoaggregation Assay

Autoaggregation was adapted from Kos et al. [26]. Initially, suspensions of lactobacilli (10^8^ CFU.mL^−1^) were vortexed (10 s) and incubated (5 h, room temperature). The absorbance (660 nm) was measured at time 0 hour (*A*_0_) and after 5 hours (*A*_5_). The percentage of autoaggregation was calculated using Equation (2).

(2) $$\begin{array}{c} \text{AA}\%=\frac{A0-A5}{A0}\times 100. \end{array}$$

#### 2.5.4. Coaggregation Assay

Coaggregation was adapted from Kos et al. [26]. Initially, cell suspensions (10^8^ CFU.mL^−1^) containing mixed suspensions containing equal volumes (1 mL) of each *Lactobacillus* and pathogens were vortexed (10 s) and incubated (4 h, 37°C). The absorbance (660 nm) was measured before and after incubation. The percentage of coaggregation was calculated using the formula below, where *A*_LAC_ indicates the absorbance of lactobacilli, *A*_PAT_ indicates the absorbance of pathogen, and *A*_MIX_ indicates the absorbance of the mixtures.

(3) $$\begin{array}{c} \text{CA}\%=\frac{\left(\left(A\text{lac}+A\text{pat}\right)/2\right)-A\text{mix}}{\left(A\text{lac}+A\text{pat}\right)/2}. \end{array}$$

### 2.6. Evaluation of Antimicrobial Activity of Lactobacilli

#### 2.6.1. Deferred Inhibition Assay

The antimicrobial activity evaluated by deferred inhibition assay was tested according to Nardi et al. [27]. Initially, an aliquot (5 *μ*L) of each lactobacilli strain suspension (10^8^ CFU.mL^−1^) was pipetted in the center of the plate with MRS agar. After incubation (48 h, 37°C, 5% CO_2_ atmosphere), colony cells were killed by exposure to chloroform (100 mL, 30 min). Residual chloroform was evaporated off, and the Petri dish overlayed with BHI semisolid agar (3.5 mL, 0.75%, *w*/*v*) previously inoculated with pathogens (1%, *v*/*v*, 10^8^ CFU.mL^−1^). After overnight incubation (18–24 h, 37°C, 5% CO_2_ atmosphere), the presence or absence of inhibition halos was observed, followed by measuring the inhibition halos (millimeters). Sterile MRS broth was considered a negative control.

#### 2.6.2. Microdiffusion Assay

The presence of diffusible inhibitory substances was also evaluated by the microdiffusion assay on semisolid agar adapted from Rodrigues et al. [28]. Initially, suspensions of pathogens (10^8^ CFU.mL^−1^) were added (1%, *v*/*v*) on semisolid BHI agar (0.75%, *w*/*v*) and plated. After solidification, sterile PVC cylinders (8 mm) were placed centrally on the plates, and inside them, aliquots (100 *μ*L) of CFCS from each *Lactobacillus* were added. After incubation (18–24 hours, 37°C, 5% CO_2_ atmosphere), the presence or absence of inhibition halos was observed, followed by measuring the inhibition halos (millimeters). Sterile MRS broth was considered a negative control.

#### 2.6.3. pH Modulation Assay by Lactobacilli

To evaluate the ability of lactobacilli to modulate the pH of the growth medium by producing organic acids, the methodology of Melgaço et al. [29] was used with modifications. The modulation of the pH of the growth medium by lactobacilli was evaluated under two conditions: isolated growth of *Lactobacillus* or growth of *Lactobacillus* in coincubation with pathogen. Initially, the microorganism suspensions (10^8^ CFU.mL^−1^) were obtained as previously described, and the pH of the MRS or MRS + BHI (*v*/*v*) broth was measured and adjusted to 6.5. Then, an aliquot of each *Lactobacillus* was added (10%, *v*/*v*) to the MRS or MRS + BHI broth. After that, the same volume of pathogens was added to the MRS + BHI broth. After incubation (24 h, 37°C, 5% CO_2_ atmosphere), the cultures were centrifuged (3,000 × *g*, 15 min); the supernatant was separated from the bacterial pellet, and the pH was measured (HMMPB-210, Highmed, Tatuapé, Brazil).

#### 2.6.4. Inhibition Assay by Coculture

The antimicrobial activity of lactobacilli against pathogens was tested by coculture assay adapted from Hütt et al. [30]. Initially, activated cultures of pathogens and lactobacilli (10^8^ CFU.mL^−1^) were inoculated together (1%, *v*/*v*) in mixed growth medium (0.5 mL BHI broth + 0.5 mL MRS broth) and were incubated overnight. Then, serial dilutions were performed, and aliquots (10 *μ*L) were seeded on blood or chocolate agar, and the plates were incubated again overnight. Cultures performed with the pathogen alone were used as negative controls. The growth of the pathogen with each *Lactobacillus* strain was compared with the growth of the control.

### 2.7. Evaluation and Characterization of the CFCS

#### 2.7.1. Amplex Red Hydrogen Peroxide Assay

Hydrogen peroxide levels present in CFCS were measured using the Amplex Red Hydrogen Peroxide/Peroxidase kit according to the manufacturer's recommendations (Thermo Fisher Scientific, Waltham, USA). After preparing the kit stock solutions, aliquots (50 *μ*L) of the standard curve samples, controls, and experimental samples were added to individual wells on a microplate. After that, the Amplex® Red reagent/HRP working solution (50 *μ*L) was added to the wells previously plotted. After incubation (30 min, room temperature, protected from light), absorbance was measured in a microplate reader (550 nm) to construct the standard curve and measure the H_2_O_2_ concentration (*μ*M) of the CFCS.

#### 2.7.2. Detection of Organic Acids, Thermotolerant Antimicrobial Substances, and Bacteriocin in the CFCS

The lactobacilli strains were assayed for production of organic acids, thermotolerant antimicrobial substances, and bacteriocins using the agar well diffusion technique described by Touré et al. [31] with modifications. Initially, *G. vaginalis* suspension (10^8^ CFU.mL^−1^) was swabbed onto 5% blood agar plates, and *N. gonorrhoeae* suspension (10^8^ CFU.mL^−1^) was swabbed onto chocolate agar plates. After that, the plates were incubated (30 min, room temperature). Concomitantly, CFCS aliquots were distributed in fractions for treatment. For the organic acid assay, the CFCS was adjusted to pH 6.5 ± 0.1 using 1 mol.L^−1^ of sodium hydroxide; for the thermotolerant substance assay, the CFCS was incubated at high temperature (5 min, 100°C), and for bacteriocin assay, the CSCF was treated with trypsin (1%, *v*/*v*; Gibco, Mississauga, Canada) or proteinase K (1%, *v*/*v*, Invitrogen, Darmstadt, Germany). Then, aliquots (100 *μ*L) of treated CFCS and untreated CFCS were added into the wells (8 mm diameter) previously made on the chocolate and 5% blood agar plates. The plates were incubated overnight, and diameters of inhibition zones (including the 8 mm-well diameter) were measured.

#### 2.7.3. Analysis of the CFCS Metabolome by GC-MS

The composition analysis of CFCS was performed by gas chromatography-mass spectrometry (GC-MS) according to the method described by Rodrigues et al. [28]. First, CFCS were lyophilized for this assay. Lyophilized CFCS were submitted to derivatization reaction by silylation. In this reaction, each sample (3 mg) was diluted in pyridine (60 *μ*L), and 100 *μ*L of *N*,*O*-Bis(trimethylsilyl) trifluoroacetamide (BSTFA) containing 1% trimethylchlorosilane was added to this solution (Sigma-Aldrich, Darmstadt, Germany). The solutions were heated (70°C, 30 min) in a water bath, and after that, an aliquot (1 *μ*L) of the diluted samples was injected into the QP2010SE-GC2010 Plus (Shimadzu, Kyoto, Japan) chromatograph with Rtx-5MS (30 m, 0.25 mm internal diameter, 0.25 *μ*m film). For chromatographic analysis, helium gas was used as the carrier gas. The temperature employed in the injector, detector, and interface GC-MS system was 290°C. The analysis of the initial temperature was 80°C (5 min), progressively increasing up to 285°C in a ratio of 4°C/minutes. The final temperature remained at 285°C (20 min). The detector mass operated ionization electron impact (70 eV), and the scan mass operated 30 to 600 Da. The identification of compounds by GC-MS was performed by comparing the mass spectra of the samples with existing spectra in the device database (NIST 08, FFNSC1.3 and WILEY8).

### 2.8. Statistical Analysis

The GraphPad Prism 6.0 software (GraphPad Software, Inc., San Diego, USA) was used for statistical analysis. Quantitative data are presented by means and standard deviations. Normality was tested by D'Agostino and Pearson, Shapiro-Wilk, and KS tests. The statistical differences between the mean values were determined by the *t*-test, Mann–Whitney test, or Kruskal-Wallis test with Dunn's posttest. Data were considered statistically significant when ∗ = *P* < 0.05, ∗∗*P* < 0.01, ∗∗∗ = *P* < 0.001, and ∗∗∗∗ = *P* < 0.0001. Except for CFCS metabolome, all assays were performed in triplicate.

## 3. Results and Discussion

### 3.1. Technological Characteristics of Lactobacilli

**Figure 1:**
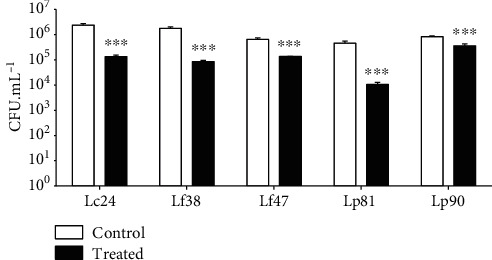
Heat resistance standard of lactobacilli isolated from cupuaçu fermentation. Lc24: *L. casei* 24; Lf38: *L. fermentum* 38; Lf47: *L. fermentum*; Lp81: *L. plantarum* 81; Lp90: *L. plantarum* 90. ^∗∗∗^Statistically significant differences compared to control (*P* < 0.001). Presented values represent the mean and the standard deviation from triplicate determinations.

Figure 1 shows the resistance of five lactobacilli strains to high temperature. According to our data, all lactobacilli tested had a significant reduction in viable cells (*P* < 0.05). Nevertheless, all *Lactobacillus* strains remained viable after the heat shock. Many species of lactobacilli are known to tolerate a rather wide temperature range, with most species capable of growing to at least 45°C [32]. The use of thermotolerant strains is advantageous for the pharmaceutical, beverage, and food industries, as their genetic features are available, adjustable, and better developed to face stressful processes [33–35]. In general, our data showed that five lactobacilli strains were considered thermotolerant, demonstrating their ability to grow at 50°C.

**Figure 2:**
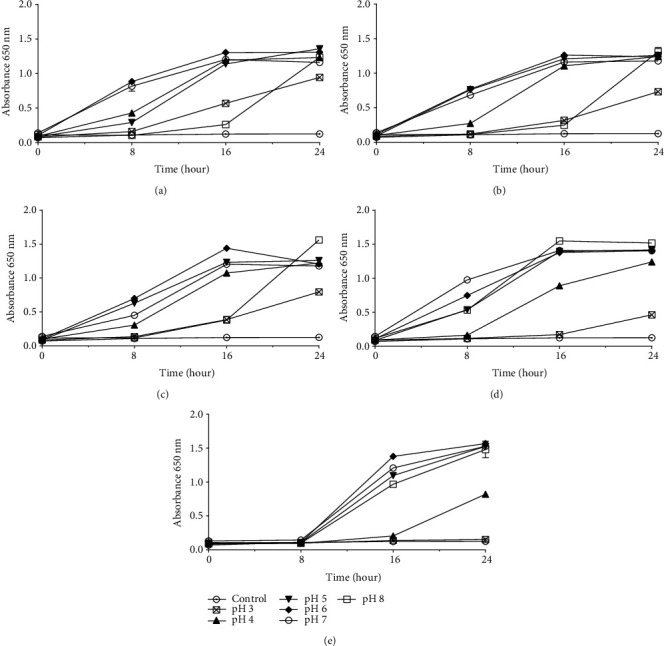
Growth of lactobacilli in different pH ranges: (a) *L. casei* 24; (b) *L. fermentum* 38; (c) *L. fermentum*; (d) *L. plantarum* 81; (e) *L. plantarum* 90. Presented values represent the mean and the standard deviation from triplicate determinations.

Our strains were also tested for their ability to survive and grow at different pH ranges, as can be seen in Figure 2. With the exception of the Lp90 strain that did not grow at pH 3, all other lactobacilli strains grew at all pHs. The resistance at pH 3 is the standard for acid tolerance of probiotic culture, and the ability of lactobacilli to adapt to the acidic environment can be influenced by growth in MRS broth [36, 37]. Using lactobacilli that grow in a wide pH range (3–8), as occurred with our strains, is of relevant and useful for intravaginal applications in patients with genital infections dominated by anaerobes that raise the pH. The introduction of lactobacilli that survive in this environment may, after adaptation, lower the pH through the production of organic acids and reduce the population of uropathogens [2].

### 3.2. Safety Assessment of Lactobacilli for Use in Humans as Probiotics

Probiotics are safe microorganisms, and nowadays, they are consumed as food, dietary supplements, and medicine all around the world. However, in recent years, many researchers have warned of an increase in antibiotic resistance in lactobacilli strains [38]. Furthermore, the WHO recommended that classifying a microorganism as a probiotic should be based on a series of tests including checking the safety of the strains, such as resistance to antibiotics and hemolytic activity [39]. Thus, we consider it necessary to guarantee the safety of our strains, since there is the possibility of transferring resistance genes from lactobacilli to pathogens or commensal bacteria [40]. In this study, the antimicrobial susceptibility profile of lactobacilli isolated from pulp of cupuaçu fruit was tested against nine antibiotics belonging to different classes (Table 1). All lactobacilli tested were resistant to vancomycin and sensitive to ampicillin, ceftriaxone, penicillin G, clindamycin, chloramphenicol, erythromycin, and nitrofurantoin.

Regarding ciprofloxacin, Lc24 and Lf47 showed resistance, and Lf38, Lp81, and Lp90 strains were classified as moderately sensitive. Resistance to quinolones (ciprofloxacin) and glycopeptides (vancomycin) exhibited by our lactobacilli strains may be related to the intrinsic resistance mechanism of this genus in relation to these antibiotics [41]. Intrinsic resistance is generally harmless when present in lactobacilli because it is not a transferable characteristic. Some authors have shown that lactobacilli may be resistant to ciprofloxacin due to the modified topoisomerase IV present in lactobacilli and that they are the main target of quinolone [42, 43]. In relation to vancomycin, lactobacilli are naturally resistant due to the presence of peptidoglycan precursors with d-alanyl-d-lactate termination that prevent the binding of the antibiotic to the cell wall [44].

Together with these data, the results in Table 2 show that all *Lactobacillus* strains were considered nonhemolytic. Previously, it was believed that this virulence factor was related only to pathogens and that lactobacilli were unable to cause hemolysis [45, 46]. However, there are reports in the literature that have demonstrated the existence of lactobacilli with hemolytic activity [47]. A study conducted by Kaktcham et al. [48], for example, described lactobacilli isolated from food and dairy products with *α*-hemolysis. Many authors, however, reported lactobacilli without hemolytic activity [48–51], similar to our data. Regarding hemolytic activity, our *Lactobacillus* strains have proven to be safe due to the absence of this characteristic.

### 3.3. Cell Surface Properties of Lactobacilli

The results of the biofilm formation and hydrophobicity of the *Lactobacillus* strains are reported in Table 2. Producing biofilm is a fundamental microbial survival mode, and the evaluation of biofilm formation and its time-dependent mechanisms are important for understanding developing therapeutic interventions and host-microbial interaction [52]. When assessing the ability of strains to form biofilm, all lactobacilli tested were biofilm producers. Our data revealed that the strains Lc24 and Lf38 were classified as moderately adherent, and the strains Lf47, Lp81, and Lp90 were considered strong biofilm producers. The types of biofilms formed by lactobacilli may be strain specific and may be related to the specific bacterial properties of the surface encoded by each genome [53]. The formation of biofilm by lactobacilli is a key factor for maintaining these microorganisms in stable in vivo in vivo ecosystems. Thus, it is clear that the formation of biofilm by beneficial strains is a desirable probiotic property, since it can promote both the colonization and the long-term presence of lactobacilli in vaginal mucosa [54].

In the present study, our lactobacilli strains showed great variation in relation to hydrophobicity, ranging from 12.56% to 85.9%. Similarly, other authors have observed great variation in the hydrophobicity of their *Lactobacillus* strains [55, 56]. According to the MATH classification, Lc24 and Lf38 showed low hydrophobicity, Lp81 showed moderate hydrophobicity, and Lf47 and Lp90 strains showed high hydrophobicity. Regarding MATS classification, Lf47 and Lp90 were considered hydrophobic, Lc24 and Lf47 were considered hydrophilic, and the Lp81 strain was considered amphiphilic. It is known that hydrophobicity is an important physicochemical property that interferes with adhesion of bacteria to host cells, which varies between strains [57, 58]. In this way, adherence is an important probiotic criterion for selecting probiotic strains, as it is also involved in the modulation of the host's immune response [59, 60]. Our data for Lf47 and Lp90 strains showed a significant increase in hydrophobicity compared to the Lc24 strain (*P* < 0.05), suggesting that these strains may remain in the vaginal mucosa longer than other lactobacilli tested.

The ability of lactobacilli to aggregate with strains of the same species was performed by the autoaggregation assay. Table 2 shows that the autoaggregation did not vary between *Lactobacillus* species (20.30% to 23.57%), except for Lf47, which presented less aggregation than the others (13%) (*P* < 0.05). In a study done by Chen et al. [61], *L. casei* and *L. plantarum* were evaluated for autoaggregation for 24 hours. Up to the time of 4 hours, all lactobacilli strains showed 7.8% to 12.8% of autoaggregation, lower values than those presented by our strains after 5 hours. However, the authors demonstrated that after 24 hours of incubation, all strains increased the percentage of autoaggregation to values between 32.12% and 47.04%, suggesting that aggregation among lactobacilli tends to increase over time. In fact, the autoaggregation capacity of lactobacilli is time-dependent and strain-specific, highlighting the importance of this mechanism as an association factor with other probiotic characteristics such as biofilm formation and mucosal adhesion [62–64].

In a similar way to autoaggregation, the direct interaction of lactobacilli strains with genital pathogens was evaluated by the coaggregation assay. The Lp90 strain showed the highest percentages of coaggregation with *G. vaginalis* and *N. gonorrhoeae* with 35.55% and 51.70%, respectively. Our data (Table 2) demonstrate that our lactobacilli were able to aggregate more efficiently with *N. gonorrhoeae* compared to *G. vaginalis* (*P* < 0.05). Coaggregation is a key event for eliminating pathogens. It has been suggested that, through this mechanism, the direct interaction of lactobacilli with pathogenic microorganisms creates an antagonistic microenvironment for pathogens, in which antimicrobial substances are secreted locally, impairing epithelial colonization [65]. Similarly, other authors have also demonstrated the ability of lactobacilli to coaggregate with *N. gonorrhoeae* [66, 67]. Other authors, however, report coaggregation data with *G. vaginalis* with a highly variable percentage, something that may indicate that the coaggregation of lactobacilli with this pathogen must be strain-specific [68, 69].

### 3.4. Detection of Inhibitory Activity of Lactobacilli

Table 3 shows the identification of antimicrobial compounds against *G. vaginalis* and *N. gonorrhoeae*. Two different assays were used to evaluate inhibiting activity of the lactobacilli strains against these genital pathogens. In the deferred inhibition assay, our results demonstrated that all lactobacilli strains were bioactive compound producers. Regarding *G. vaginalis*, the substances produced by lactobacilli formed halos, varying from 14.33 to 21.00 mm. With respect to *N. gonorrhoeae*, the compounds secreted by lactobacilli formed larger inhibition halos in relation to the first pathogen, ranging from 20.67 to 31.67 mm in diameter. In another similar study, Pessoa et al. [21] evaluated three strains of lactobacilli isolated from cocoa fermentation against *G. vaginalis* ATCC 49154 (same strain of this study) and reported halos with diameters ranging from 11 to 12 mm using the agar diffusion assay.

In the second assay, microdiffusion showed that the bioactive compounds inhibited the growth of pathogens by contact or by the presence of halos. The strains Lc24, Lf38, Lf47, and Lp81 inhibited the growth of genital pathogens by contact. Only the Lp90 strain exhibited inhibition halo by this methodology. The halo of inhibition against *G. vaginalis* was larger (17.33 mm) than the halo presented against *N. gonorrhoeae* (13.67 mm). Coincidentally, Qian et al. [70] also applied two methodologies similar to the tests carried out in this study to identify the presence of antimicrobial compounds. According to the authors, the five tested *Lactobacillus* strains displayed the ability to inhibit *G. vaginalis*, and the halo formation was higher in the deferred assay (10.00–13.67 mm) compared to microdiffusion (1.00–3.02 mm). In another study, Carmo et al. [66] evaluated seven reference strains of *Lactobacillus* against three genital pathogens, including *N. gonorrhoeae*. Six of these lactobacilli showed antimicrobial activity with zones of inhibition ranging from 9.0 to 19.3 mm in diameter. Our results agree with these findings, because all supernatants presented antipathogenic activity; however, larger inhibition diameters were found in the present study.

### 3.5. Antimicrobial Properties of Lactobacilli in Coculture with *G. vaginalis* and *N. gonorrhoeae*

**Figure 3:**
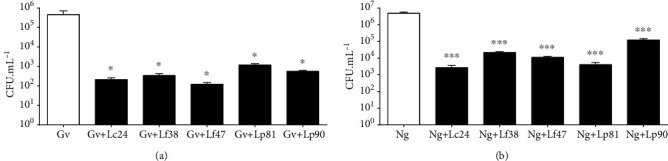
Growth inhibition of pathogens by lactobacilli after 24 h of culture. The growth of pathogenic microorganism is expressed as log_10_ CFU.mL^−1^. Each value corresponds to the mean ± standard deviation of triplicate determinations. Control: (Gv) *G. vaginalis* or (Ng) *N. gonorrhoeae*. The different lactobacilli isolates are represented by their respective numbers: (a) represents coculture of *G. vaginalis* with lactobacilli; (b) represents coculture of *N. gonorrhoeae* with lactobacilli. ^∗^Statistically significant differences compared to control (*P* < 0.05). ^∗∗∗^Statistically significant differences compared to control (*P* < 0.001). Presented values represent the mean and the standard deviation from triplicate determinations.

The pH of isolated culture of lactobacilli and coculture of *Lactobacillus* strains with pathogen was measured, and the data are shown in Table 3. There was no significant difference (*P* > 0.05) between the pH of *Lactobacillus* isolated culture and cocultures. This demonstrates that lactobacilli are able to reduce the pH of the culture (initial pH of 6.5) and keep it low, even with the presence of genital pathogens. In this study, the highest pH was from the coculture of Lf38 with diplococcus (4.68), and the lowest pH recorded was from Lf90 with diplococcus (3.75). In our study, we also evaluated the inhibitory effect of *Lactobacillus* on the growth of pathogens in coculture (Figure 3). Noting the inhibition caused by a direct interaction between our lactobacilli strains and pathogens, we observed that the growth of *G. vaginalis* and *N. gonorrhoeae* decreased compared to the control. In relation to *G. vaginalis*, all lactobacilli tested were able to decrease its growth (*P* < 0.05). In the coculture with *N. gonorrhoeae*, all lactobacilli tested also decreased the growth of diplococcus after 24 hours (*P* < 0.001).

The acidification potential of lactobacilli under anaerobic conditions is an important characteristic conferring protection against pathogens, such as *N. gonorrhoeae* [71]. Considering our data, we also believe that the reduction in pH promoted by the secretion of organic acids by our lactobacilli strains constituted a fundamental mechanism for inhibiting the growth of *N. gonorrhoeae* and *G. vaginalis*. According to our study, Bertuccini et al. [72] demonstrated that *Lactobacillus acidophilus* and *Lactobacillus rhamnosus* were able to inhibit the growth of *Staphylococcus aureus*, *Escherichia coli*, *Atopobium vaginae*, and *G. vaginalis* at different incubation times. We demonstrated that our *Lactobacillus* strains are potential probiotic candidates because they were able to maintain a low pH and fight the growth of the pathogens tested, corroborating the literature that points to the genus *Lactobacillus* as a potential source of bacteria with safe probiotic characteristics [73].

### 3.6. Production of H_2_O_2_ by Lactobacilli

**Figure 4:**
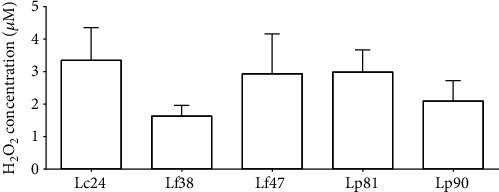
Hydrogen peroxide levels in CFCS of lactobacilli isolated from cupuaçu fermentation. The standard curve (*R*^2^ = 0.9927) was performed together with the experimental samples in a controlled environment protected from light. Presented values represent the mean and the standard deviation from triplicate determinations.

Figure 4 demonstrates the production of hydrogen peroxide by lactobacilli isolated from cupuaçu pulp. In this study, all five lactobacilli tested were able to produce hydrogen peroxide, and the H_2_O_2_ concentration varied between 1 and 5 *μ*M. There was no significant difference (*P* > 0.05) between strains or species studied. Classically, lactobacilli are considered biomarkers of women's health, as they protect vaginal mucosa against genital infections due to the presence of lactic acid and H_2_O_2_ [74–76]. It is known that hydrogen peroxide-producing lactobacilli are commonly isolated from the healthy vagina of women of childbearing age, which may make this type of *Lactobacillus* an important element in maintaining genital homeostasis [77], since the presence of these microorganisms reduces the risk of BV and infections associated with BV [78]. The literature suggests that the main advantages associated with H_2_O_2_ activity are its ability to cross the cell membrane of the pathogen, to damage the membrane, proteins, DNA, and enzymes by the formation of free radicals and the anti-inflammatory stimulus of the vaginal mucosa [79–81]. Furthermore, the joint action of metabolites, such as H_2_O_2_, organic acids, bacteriocins, and biosurfactants, has already been attributed as a potent antibiotic-assisting tool to contain genital infections and minimize resistance to antibiotics [82].

### 3.7. Characterization of Antimicrobial Substances in CFCS

After verifying that our five lactobacilli strains showed antimicrobial activity against pathogens, we identified these substances. Treatments were applied with the CFCS responsible for inhibiting pathogens in the previous tests. The treatments of CFCS included neutralization of possible organic acids, boiling, and inactivation of possible bacteriocins through the enzymatic action of trypsin and proteinase K. Our results are shown in Table 4. Our data showed that only CFCS from *L. plantarum* (Lp81 and Lp90) inhibited the growth of *G. vaginalis* when untreated or treated by boiling or enzymatic action. The neutralized CFCS allowed the growth of the pathogen. Regarding *N. gonorrhoeae*, only the CFCS of the Lf38 and Lp90 strains were able to inhibit the bacterial growth of the diplococcus. The inhibition and growth profile of these bacteria were similar to that of the first. CFCS treated with trypsin or proteinase K did not affect the growth of both bacteria, suggesting that the compound for the antibacterial activity is not related to the class of bacteriocins. We inferred from our data that the bioactive compounds present in the CFCS responsible for the antimicrobial action probably are the organic acids. This is explained by the fact that bacterial growth was only possible when the CFCS was neutralized. Also, we realized that organic acids were thermostable compounds, since there was no growth of pathogens after boiling.

The analysis of the CFCS metabolome of our lactobacilli strains demonstrated a wide variety of organic compounds. Among the main substances, there were organic acids, alcohols, sugars, and other biological compounds (Table 5). In this study, however, we evaluated only the profile of organic acids produced by five lactobacilli strains, and the CFCS characterization revealed that lactic acid was the most prevalent organic acid in the sample, ranging from 49.18% to 61.77% of the analyte. The secretion of organic acids was quite diverse, even within the same species. In this study, we conclude that the types of acids produced are strain-specific and are not species-specific. Other organic acids were also produced by the strains, including acetic acid, butyric acid, 4-methyl-2-hydroxypentanoic acid, 3-methyl-2-hydroxypentanoic acid, malic acid, 2-pyrrolidone-5-carboxylic acid, benzenepropanoic acid, and pentanedioic acid. We also believe that strain-specific production of organic acids is responsible for the different patterns of inhibition presented against *G. vaginalis* and *N. gonorrhoeae*.

Lactic acid plays a complex and critical role in maintaining of homeostasis in the healthy vaginal environment, because it is believed that an important function of lactic acid is inhibiting the growth of genital pathogens, such as *G. vaginalis* [83]. Many studies have reported that lactic acid produced by lactobacilli favors the promotion of vaginal health and has been shown to inhibit the growth of pathogenic organisms in vitro [84, 85] The literature indicates that women with a predominance of lactobacilli in the vaginal microbiota are less susceptible to infection by *N. gonorrhoeae* [86]. Besides that, many studies have shown that a complex vaginal microenvironment with lactic acid and hydrogen peroxide inhibits genital pathogens, such as *N. gonorrhoeae* [67, 71, 87]. Finally, we understand that the growth of genital pathogens depends on the pH, and the production of lactic acid by our lactobacilli strains must be sufficient to reduce the pH and thus inhibit the growth of anaerobes [88].

## 4. Conclusion

The current understanding of the complex interaction between lactobacilli, genital pathogens, and vaginal mucosa of the human host remains incomplete. Our strains proved to be promising probiotic candidates due to the different characteristics analyzed. All five *Lactobacillus* strains isolated from Brazilian cupuaçu were considered safe for use in humans according to trials recommended by the WHO. In general, our strains were resistant to temperature and pH stresses, desirable requirements for strains targeted in the industry. Besides this, the five lactobacilli strains were biofilm producers and inhibited the growth of two pathogens in different methodologies. In addition, our strains produce H_2_O_2_ and are able to reduce the pH of the medium through the production of organic acids, especially lactic acid, an important mechanism to combat genital pathogens. Collectively, our data demonstrated that our lactobacilli strains are promising probiotic candidates. We understand that tests on living models will be necessary to confirm our findings and, possibly, to develop mixed or separate products of lactobacilli and supernatants in order to contribute to vaginal homeostasis. In the future, we believe that our strains can be applied as probiotics usable in clinical conditions.

## Figures and Tables

**Table 1:** Susceptibility profile of five lactobacilli isolated strains from cupuaçu fermentation.

Antimicrobial			Susceptibility				
Type	Name	Disc contents	Lc24	Lf38	Lf47	LP81	Lp90
Inhibitors of cell wall synthesis	Ampicillin	10 *μ*g	S	S	S	S	S
	Ceftriaxone	30 *μ*g	S	S	S	S	S
	Penicillin G	10 *μ*g	S	S	S	S	S
	Vancomycin	30 *μ*g	R	R	R	R	R
Inhibitors of protein synthesis	Clindamycin	2 *μ*g	S	S	S	S	S
	Chloramphenicol	30 *μ*g	S	S	S	S	S
	Erythromycin	15 *μ*g	S	S	S	S	S
Inhibitors of nucleic acid synthesis	Ciprofloxacin	5 *μ*g	R	MS	R	MS	MS
Other urinary tract antiseptics	Nitrofurantoin	300 *μ*g	S	S	S	S	S

Susceptibility expressed as sensitive (S), moderately sensitive (MS), or resistant (R).

**Table 2:** Hemolytic activity and characterization of physiological and antimicrobial properties of five lactobacilli strains isolated from cupuaçu fermentation.

Strain	Hemolytic activity	Biofilm formation	Hydrophobicity (%)	Autoaggregation (%)	Coaggregation (%)	
					*G. vaginalis*	*N. gonorrhoeae*
Lc24	*γ*-Hemolytic	Moderately adherent	12.56 ± 1.11^a^	25.18 ± 0.72^a^	18.25 ± 0.73^a1^	36.82 ± 2.40^a2^
Lf38	*γ*-Hemolitic	Moderately adherent	28.03 ± 0.76^b^	23.33 ± 1.93^a^	15.83 ± 1.44^b1^	32.11 ± 1.53^b2^
Lf47	*γ*-Hemolitic	Strongly adherent	85.90 ± 2.91^c^	13.13 ± 0.44^b^	19.66 ± 0.83^a1^	39.71 ± 0.00^a2^
Lp81	*γ*-Hemolitic	Strongly adherent	40.29 ± 1.25^d^	20.30 ± 1.30^a^	14.93 ± 2.24^b1^	36.13 ± 3.53^a2^
Lp90	*γ*-Hemolitic	Strongly adherent	81.54 ± 2.78^c^	23.57 ± 0.72^a^	35.55 ± 2.98^c1^	51.70 ± 2.26^c2^

Presented values are means of triplicate determinations; ± indicates standard deviations from the mean. Mean values (±standard deviation) of coaggregation within the same column followed by different superscript letters differ significantly (*P* < 0.05). Mean values (±standard deviation) of coaggregation within the same line followed by different superscript numbers differ significantly (*P* < 0.05).

**Table 3:** Antimicrobial evaluation of bioactive compounds produced by five lactobacilli strains isolated from cupuaçu fermentation.

Strain/broth	Deferred inhibition (mm)		Microdiffusion (mm)		pH		
	*G. vaginalis*	*N. gonorrhoeae*	*G. vaginalis*	*N. gonorrhoeae*	Isolated growth	Coincubation (Gv)	Coincubation (Ng)
Lc24	15.00 ± 0.00	26.33 ± 0.58	Contact	Contact	4.38 ± 0.09^a1^	4.23 ± 0.02^a1^	4.48 ± 0.01^a1^
Lf38	15.67 ± 1.53	20.67 ± 1.16	Contact	Contact	4.37 ± 0.13^a1^	4.20 ± 0.02^a1^	4.68 ± 0.27^b1^
Lf47	14.33 ± 1.16	25.00 ± 0.00	Contact	Contact	4.37 ± 0.13^a1^	4.26 ± 0.01^a1^	4.37 ± 0.15^a1^
Lp81	21.00 ± 0.00	23.33 ± 3.51	Contact	Contact	3.93 ± 0.21^b1^	4.04 ± 0.04^b1^	4.13 ± 0.03^c1^
Lp90	21.00 ± 0.00	31.67 ± 2.89	17.33 ± 0.58	13.67 ± 0.58	3.78 ± 0.05^b1^	3.85 ± 0.02^c1^	3.75 ± 0.02^d1^
MRS	0.00 ± 0.00	0.00 ± 0.00	0.00 ± 0.00	0.00 ± 0.00	6.50 ± 0.00^c1^	6.50 ± 0.00^d1^	6.50 ± 0.00^e1^

Presented values of pH assay are means of triplicate determinations; ± indicates standard deviations from the mean. Mean values (± standard deviation) within the same column followed by different superscript letters differ significantly (*P* < 0.05). Mean values (±standard deviation) within the same line followed by different superscript numbers differ significantly (*P* < 0.05). Gv: *G. vaginalis*; Ng: *N. gonorrhoeae*. The measurement of the inhibition halos of the microdiffusion assay includes the diameter of the PVC cylinder (8 mm). Contact means that there was no growth of the pathogen just inside the PVC cylinder.

**Table 4:** Inhibitory activity of treated and untreated CFCS of *Lactobacillus* strains against *G. vaginalis* and *N. gonorrhoeae.*

Strain	MRS	Untreated CFCS	Neutralized CFCS	Boiled CFCS	CFCS + trypsin	CFCS + proteinase K
	Growth of *G. vaginalis*					
Lc24	+	+	+	+	+	+
Lf38	+	+	+	+	+	+
Lf47	+	+	+	+	+	+
Lp81	+	Inhibited	+	Inhibited	Inhibited	Inhibited
Lp90	+	Inhibited	+	Inhibited	Inhibited	Inhibited
	Growth of *N. gonorrhoeae*					
Lc24	+	+	+	+	+	+
Lf38	+	Inhibited	+	Inhibited	Inhibited	Inhibited
Lf47	+	+	+	+	+	+
Lp81	+	+	+	+	+	+
Lp90	+	Inhibited	+	Inhibited	Inhibited	Inhibited

(+) means growth of pathogen.

**Table 5:** Metabolomic analysis of CFCS.

Retention time (min)	Substance	MRS area (%)	CFCS area (%)	CFCS area (%)	CFCS area (%)	CFCS area (%)	CFCS area (%)
			Lc24	Lf38	Lf47	Lp81	Lp90
4.164	Carbodiimide	—	1.48	0.74	0.89	1.51	1.37
4.700	N.N-Dimethylglycine	0.04	—	0.03	—	0.08	—
7.295	Lactic acid	0.85	61.77	49.18	56.43	57.64	61.05
7.524	Acetic acid	—	—	0.05	0.07	—	0.04
7.531	2-Hydroxyetanoic acid	—	—	—	—	0.04	—
7.768	Valine	0.05	—	—	—	—	—
8.405	Alanine	1.14	2.15	4.09	3.40	2.24	2.00
9.020	Glycine	0.33	0.81	1.11	0.82	0.99	0.77
9.873	*β*-Lactate	—	—	0.08	0.10	0.06	0.07
10.168	Leucine	0.17	—	—	—	—	—
10.445	3-Hydroxybutyric acid	—	—	—	—	0.04	—
10.638	*α*-Hydroxyvaleric acid	—	0.17	—	—	0.10	0.16
12.465	Valine	1.02	1.71	2.85	2.95	2.12	2.06
13.203	4-Methyl-2-hydroxypentanoic acid	—	0.35	0.87	0.71	0.29	0.58
13.395	3-Methyl-2-hydroxypentanoic acid	—	—	—	—	0.07	0.10
14.510	Leucine	2.48	2.55	3.45	4.49	3.50	3.43
14.733	Glycerol	—	0.71	—	1.34	—	—
14.769	Phosphoric acid	7.29	7.56	11.13	9.73	7.85	7.86
15.236	Isoleucine	1.18	1.89	2.65	2.74	2.17	2.15
15.308	*γ*-Amino butyric acid	—	—	0.10	—	0.14	—
15.839	Butanoic acid	0.20	0.28	0.97	1.00	0.46	0.50
16.220	2-Methyl-2.3-dihydroxypropanoic acid	0.10	0.13	0.27	0.36	0.15	0.16
16.705	Pyrimidine	—	—	0.13	—	—	—
17.644	Serine	0.75	0.85	2.23	1.19	0.61	0.47
18.148	Butanoic acid	0.02	0.12	0.09	0.11	0.06	0.07
18.272	3-Methyl-1.4-dihydroxypiperazine-2.5-dione	—	0.79	0.08	—	0.07	—
18.496	Butyric acid	—	—	2.07	—	—	—
18.531	Threonine	0.62	—	—	1.71	1.38	1.24
18.555	Lactic acid dimer	—	0.52	—	—	0.25	0.27
19.332	2.4-Dihydroxybutanoic acid	—	—	0.12	0.15	0.07	0.07
19.494	Aspartic acid	—	1.04	—	0.64	—	0.64
21.091	Trisiloxane	—	0.11	0.15	0.15	0.11	0.13
21.769	Malic acid	—	0.12	—	—	0.10	0.24
21.865	2-Pyrrolidone-5-carboxylic acid	—	0.34	—	0.58	—	0.81
22.262	Glutamic acid	—	—	—	0.15	—	—
22.458	Methionine	0.22	—	0.27	—	0.31	—
22.588	Proline	1.40	1.17	3.55	0.84	2.20	1.52
22.708	Aspartic acid	0.56	—	0.63	—	0.90	0.20
23.099	Phenylalanine	—	0.92	—	1.29	—	0.59
24.376	Benzenepropanoic acid	—	0.09	0.57	0.45	0.28	0.50
25.371	Ornithine	—	—	—	—	0.21	—
25.583	Glutamine	1.76	—	3.81	—	3.06	1.40
26.434	Tartaric acid	0.34	0.40	0.67	0.47	0.49	0.54
26.995	Hydroxy 4-oxo-2.4-di(hydroxylamine)butanoate	—	—	—	—	0.16	—
27.048	Asparagine	0.04	—	0.36	—	—	—
27.895	Lysine	—	—	1.22	—	1.11	—
28.638	Arabinitol	—	—	0.10	—	—	—
28.805	Ribitol	—	—	—	—	0.11	0.12
29.803	2.3-Dihydroxypropylphosphoric acid	0.13	0.11	0.30	0.26	0.26	0.25
29.920	D-Ribo-Hexonic acid	—	—	—	—	0.09	0.11
30.946	2-Keto-D-gluconic acid	0.7	—	—	—	0.06	—
31.133	1,2,3-Propanetricarboxylic acid	—	6.15	0.11	—	—	—
31.259	Citric acid	7.49	—	—	—	6.66	5.91
32.345	Pentanedioic acid	—	0.34	0.75	0.16	0.29	—
32.451	Benzenepropanoic acid	0.10	—	—	—	—	—
32.879	4-Hydroxyphenyllactic acid		—	—	0.07	0.10	0.16
33.891	Tyrosine	0.13	—	—	—	—	0.40
33.955	Glucitol	0.04	0.16	3.65	2.76	—	
35.660	Inositol	31.07	—	0.07	—	—	0.07
39.845	Tryptophan	0.06	—	0.26	—	0.16	—
—	Sugars	38.12	3.38	4.07	2.96	0.83	1.54
—	Unidentified compounds	1.60	1.81	1.20	3.83	0.62	0.46
—	Identified compounds (except sugars)	60.28	94.81	94.73	93.21	98.55	98.00
—	Total	100.00	100.00	100.00	100.00	100.00	100.00

## Data Availability

The datasets used and/or analyzed during the current study are available from the corresponding author on reasonable request.
